# The RECK tumor-suppressor protein binds and stabilizes ADAMTS10

**DOI:** 10.1242/bio.033985

**Published:** 2018-10-15

**Authors:** Tomoko Matsuzaki, Hitoshi Kitayama, Akira Omura, Emi Nishimoto, David B. Alexander, Makoto Noda

**Affiliations:** 1 Department of Molecular Oncology, Kyoto University Graduate School of Medicine, Yoshida-Konoe-cho, Sakyo-ku, Kyoto 606-8501, Japan; 2 Department of Molecular Toxicology, Nagoya City University, Graduate School of Medical Sciences, 1 Kawasumi, Mizuho-cho, Mizuho-ku, Nagoya 467-8601, Japan

**Keywords:** RECK, ADAMTS10, Tumor suppressor, Fibronectin, MT1-MMP, Yeast two-hybrid assay

## Abstract

The tumor suppressor protein RECK has been implicated in the regulation of matrix metalloproteinases (MMPs), NOTCH-signaling and WNT7-signaling. It remains unclear, however, how broad the spectrum of RECK targets extends. To find novel RECK binding partners, we took the unbiased approach of yeast two-hybrid screening. This approach detected ADAMTS10 as a RECK-interactor. ADAMTS10 has been characterized as a metalloproteinase involved in fibrillin-rich microfibril biogenesis, and its mutations have been implicated in the connective tissue disorder Weill-Marchesani syndrome. Experiments *in vitro* using recombinant proteins expressed in mammalian cells indicated that RECK indeed binds ADAMTS10 directly, that RECK protects ADAMTS10 from fragmentation following chemical activation and that ADAMTS10 interferes with the activity of RECK to inhibit MT1-MMP. In cultured cells, RECK increases the amount of ADAMTS10 associated with the cells. Hence, the present study has uncovered novel interactions between two molecules of known clinical importance, RECK and ADAMTS10.

This article has an associated First Person interview with the first author of the paper.

## Abstract (precis)

**Summary:** RECK plays critical roles in tumor suppression and embryogenesis, while its mechanisms of actions remain largely obscure. This study has revealed the novel interaction between RECK and ADAMTS10.

## INTRODUCTION

*RECK* was initially isolated as a transformation suppressor gene against the v-*K-ras* oncogene (Takahashi et al., 1998); subsequent studies implicated RECK in suppression of tumor growth, angiogenesis, invasion, metastasis and recurrence (Hill et al., 2011; Noda and Takahashi, 2007; Oh et al., 2001; Yoshida et al., 2012). *RECK* is conserved from insects to mammals as a single gene. Human *RECK* encodes a glycosylphosphatidylinositol (GPI)-anchored glycoprotein of ∼125 kDa with a weak matrix metalloproteinase (MMPs) inhibitory activity (Miki et al., 2007; Oh et al., 2001; Omura et al., 2009; Takahashi et al., 1998). *Reck*-deficient mice die around embryonic day 10.5 (E10.5) with reduced tissue integrity, arrested vascular development (Oh et al., 2001) and precocious neuronal differentiation (Muraguchi et al., 2007). Some of these phenotypes have been attributed to increased proteolysis (Oh et al., 2001), attenuated Notch-signaling (Muraguchi et al., 2007) and reduced WNT7-signaling (Noda et al., 2016; Vanhollebeke et al., 2015); however, it remains unclear to what extent these mechanisms explain the *Reck* mutant phenotypes. Tissue inhibitors of metalloproteinases (TIMPs) are known as a prototypic family of endogenous MMP inhibitors which are structurally unrelated to RECK. Mice lacking all TIMPs (quadruple mutant) are viable (Shimoda et al., 2014), demonstrating distinct physiological roles for TIMPs and RECK.

ADAMTS (a disintegrin-like and metalloprotease with thrombospondin motifs) superfamily of secreted proteins consists of 19 zinc metalloproteinases and 7 ADAMTS-like proteins lacking catalytic activity. A hallmark of ADAMTS proteases is an ancillary domain containing one or more thrombospondin type 1 repeats (TSR1). Known functions of ADAMTS proteases include maturation of procollagen (ADAMTS2, 3 and 14) and von Willebrand factor (ADAMTS13) as well as extracellular matrix (ECM) cleavage involved in morphogenesis (nematode ADAMTS proteins), angiogenesis (ADAMTS1 and 9), ovulation (ADAMTS1), cancer (ADAMTS4, 5, 9 and 15) and arthritis (ADAMTS5) (Apte, 2009; Hubmacher and Apte, 2015). The first biochemical study of ADAMTS10 by Somerville et al. (2004) indicated that the gene is widely expressed in mouse embryos particularly in mesenchymal cells, that zymogen as well as processed forms of ADAMTS10 are secreted from the cells, and that substantial amounts of these protein species are retained in the pericellular region by interaction with either the cell surface or pericellular matrix components. Most ADAMTS proteases contain a consensus furin recognition and cleavage sequence (R-X-R/K-R↓) at the junction of the propeptide and catalytic domain, but ADAMTS10 diverges at this site (i.e. GLKR^233^) and lacks a sequence required for efficient cleavage by furin and other members of the subtilisin/kexin-like proprotein convertase (PC) family (Duckert et al., 2004), making it likely that ADAMTS10 is resistant to activation by all PCs and activated by other means. The discoveries that a rare connective tissue disorder, Weill-Marchesani syndrome, was genetically linked with recessive mutations of ADAMTS10 (WMS1; OMIM #277600) (Dagoneau et al., 2004) as well as dominant mutations of fibrillin-1 (WMS2; OMIM #608328) (Faivre et al., 2003) suggested that ADAMTS10 and fibrillin-1 (FBN1) might interact and/or act cooperatively in a common pathway.

In the present study, we performed yeast two-hybrid screening to find clues to the additional molecular functions of RECK, and this led to the detection of ADAMTS10 as a RECK-interactor. Experiments with purified recombinant proteins and in cultured cells also yielded evidence indicating physical and functional interactions between these molecules of known, but distinct, clinical importance.

## RESULTS

### RECK binds ADAMTS10

**Fig. 1.**
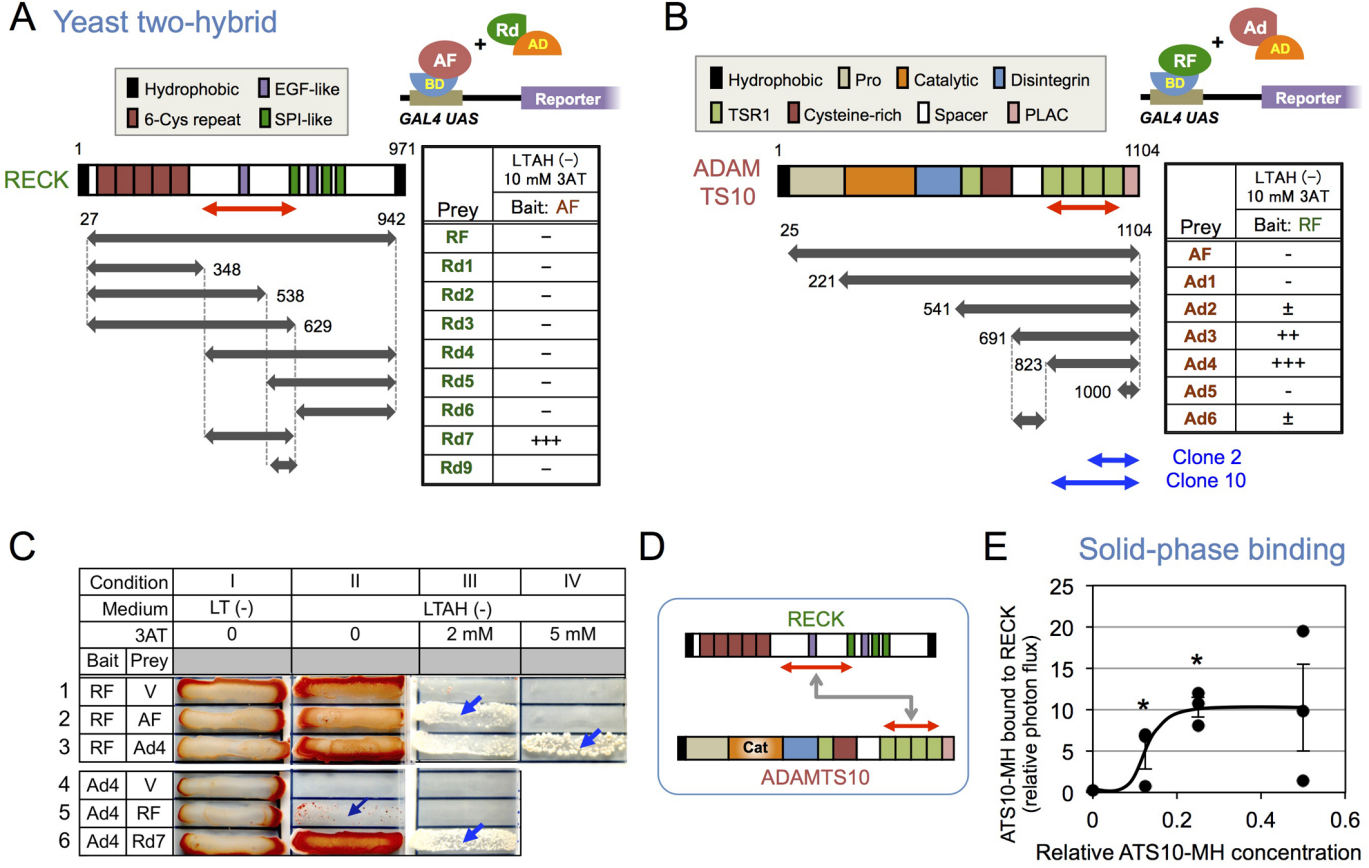
**RECK binds ADAMTS10.** (A) Interactions between ADAMTS10 and various fragments of RECK. Yeast two-hybrid assays were performed using near full-length ADAMTS10 (AF) as bait and the near full-length RECK (RF) or one of its fragments (Rd1-9) as prey. Structural features of RECK are shown in the top-left diagram. SPI, Kazal type serine protease inhibitor. The experimental setting is shown in the top-right diagram. BD: DNA-binding domain. AD: transactivation domain. (B) Interactions between RECK and various fragments of ADAMTS10. Yeast two-hybrid assays were performed using RF as bait and AF or one of its fragments (Ad1-6) as prey. Structural features of ADAMTS10 are shown in the top left diagram. TSR, thrombospondin type 1 repeat; PLAC, protease and lacunin. The experimental outline is shown in the top right diagram. In A and B, the growth (number of colonies) on selective plates is presented as follows: −, no colonies; +, a few colonies; ++, more than 50% of the control (colonies on -LT plate); +++, more than 90% of the control. The regions contained in the original two clones (2 and 10) isolated in library screening (see Table S1) are also shown (bottom). (C) Two-hybrid assays under reduced stringency. Yeast cells transformed with a plasmid expressing a bait (RF or Ad4) were further transformed with a vacant vector (V) or the vector expressing a prey (AF, Ad4, RF or Rd7) and selected with SD medium lacking two amino acids, leucine and tryptophan (LT). The selected colonies were cultured in liquid SD medium lacking LT [termed LT (−) medium] overnight, and an equal amount of yeast suspension was plated on agar plates of either LT (−) medium (Condition I), SD medium lacking adenine, histidine, leucine and tryptophan [LTAH (−) medium] (Condition II), LTAH (−) medium supplemented with 2 mM 3AT (Condition III) or 5 mM 3AT (Condition IV) followed by incubation at 30°C for 7 days. Growth under Condition I indicates the presence of two plasmids (bait and pray, respectively), while growth on other media indicates interactions between the bait and pray with weak (II) intermediate (III) and strong affinities (IV). Note the higher background when RF was used as bait (II-1). (D) Summary of the results shown in A–C. (E) Dose-dependent binding of ATS10-MH to immobilized RECK-His. See ‘Solid-phase binding assay’ in Materials and Methods for details. *P<0.05 against blank wells (non-coated dish; *n*=3 wells). Similar results were obtained in three independent experiments.

In an attempt to find novel binding partners for RECK, we used the yeast two-hybrid assay, with near-full-length RECK as bait, to screen a mouse embryo cDNA library. Among the 14 positive clones isolated and sequenced (Table S1), two independent clones (clones 2 and 10) were found to encode overlapping, COOH-terminal portions (129 and 251 amino acid residues, respectively) of ADAMTS10 (see the bottom of Fig. 1B for their positions).

To map the domains responsible for this interaction, we constructed a series of deletion mutants of both RECK (Fig. 1A, Rd1-9) and ADAMTS10 (Fig. 1B, Ad1-6) and tested their interactions again using the yeast two-hybrid assay. When the near full-length ADAMTS10 (AF: residues 25-1104) was used as bait (Fig. 1A), the RECK fragment Rd7 (residues 349-629) was strongly positive, but full length RECK (RF) and the other RECK fragments were negative under these stringent conditions: namely, LTAH (−)+10 mM 3AT (see Materials and Methods for details). When the near full-length RECK (RF: residues 27-942) was used as bait (Fig. 1B), ADAMTS10 fragment Ad4 (residues 823-1104) was strongly positive and Ad3 (residues 691-1104) was also clearly positive. While in the settings shown in Fig. 1A and B, the interaction between AF and RF was undetectable (Fig. 1A,B, top row in the tables), under less stringent conditions (condition III in Fig. 1C) their interaction was readily detected when we used RF as bait and AF as prey (Fig. 1C, row 2). Likewise, the interaction between two small fragments, Ad4 (bait) and Rd7 (prey), could be detected (Fig. 1C, row 6). These results implicate a central region of RECK and a C-terminal region of ADAMTS10 in this interaction (Fig. 1D).

To verify the interaction more directly, we expressed a full-length ADAMTS10 protein tagged with C-terminal tandem MYC and His_6_ epitopes in the mammalian cultured cell line HEK293 and purified this protein, termed ATS10-MH, from the culture supernatant (Fig. S1A,C). We also raised antibodies (termed pAb-867) against an epitope (residues 867-879) in the second TSR1 (Fig. S1A, arrow); they recognize both mouse and human ADAMTS10. Using these reagents, together with a recombinant RECK protein previously described (termed RECK-His; Fig. S1B,D) (Omura et al., 2009), we could detect the direct binding between ATS10-MH and RECK-His in a solid-phase binding assay in the presence of three cations: Ca^2+^, Mg^2+^ and Zn^2+^ (Fig. 1E). Surface plasmon resonance assay was also used to confirm this binding and to reveal that the binding requires Zn^2+^ (Fig. S1E,F).

### RECK co-localizes with ADAMTS10

**Fig. 2.**
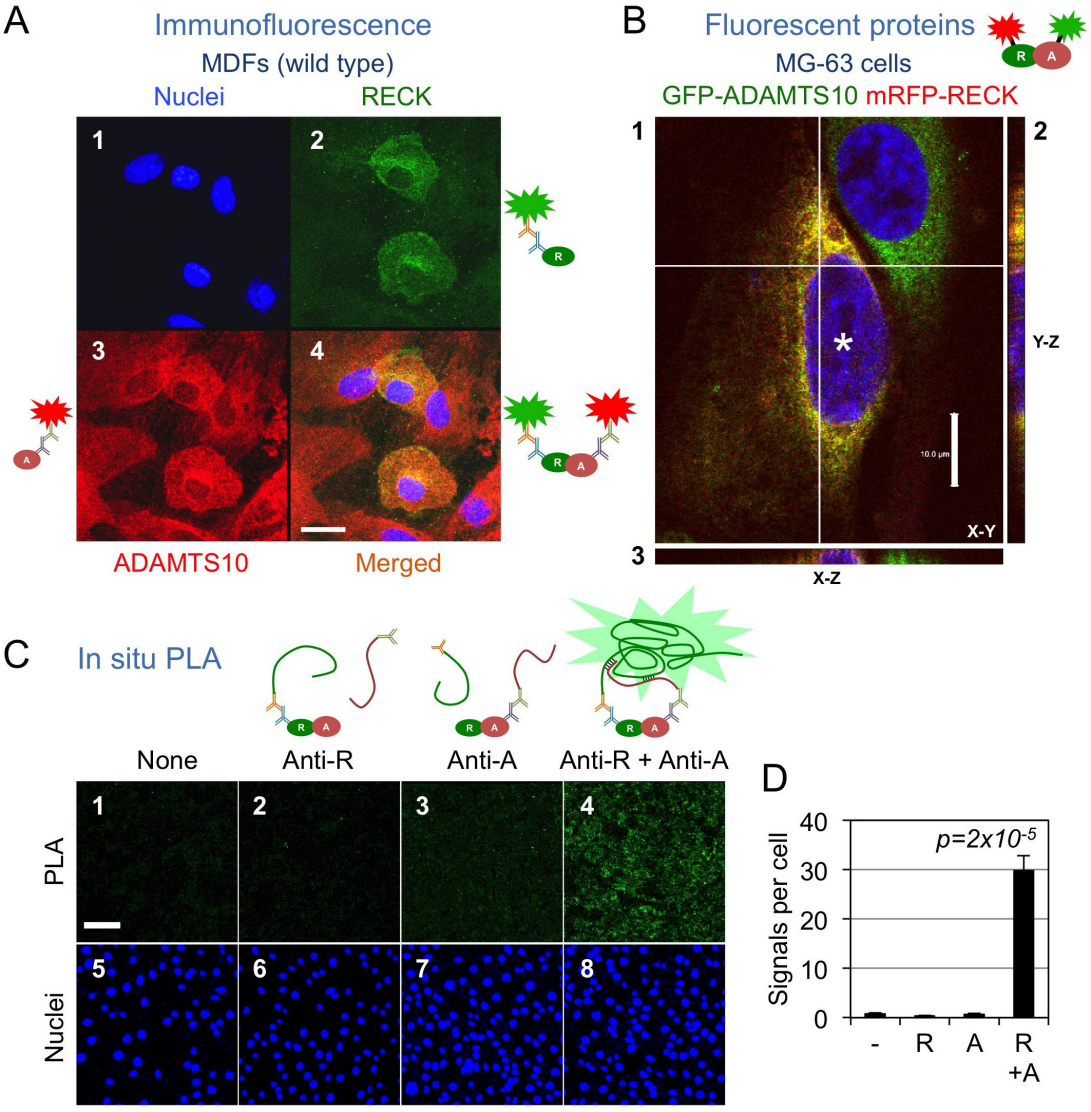
**RECK co-localizes with ADAMTS10.** (A) Immunofluorescence staining for endogenous ADAMTS10 (red, panel 3) and RECK (green, panel 2) in wild-type MDFs. Nuclei were counterstained with Hoechst-33342 (blue, panel 1). A merged image is shown in panel 4. Scale bar: 20 µm. (B) Co-expression of mRFP-RECK and GFP-ADAMTS10 in MG-63 cells. A confocal image (panel 1) and the Y-section (panel 2) and X-section (panel 3) images reconstituted from the Z-series images are shown. Scale bar: 10 µm. Asterisk: a cell expressing both proteins. (C,D) *In situ* proximity ligation assays (PLA) using antibodies against RECK (anti-R) and ADAMTS10 (anti-A) to detect close co-localization between endogenous ADAMTS10 and RECK proteins in/around NIH3T3 cells. (C) Typical results of PLA. The PLA signals were detected in the presence of the indicated antibodies (green, upper panels) and recorded after nuclear counterstaining (blue, lower panels). Scale bar: 100 µm. (D) The intensity of the PLA signals and the number of nuclei were quantified from images, as shown in C, to calculate the signal intensity per cell (*n*=8). Conceptual experimental strategy is illustrated near each panel.

Totally separate subcellular localization may preclude the physical interaction between RECK and ADAMTS10 at the cellular level. To test this possibility, we performed two types of double-labeling experiments. First, immunofluorescence staining of endogenous RECK (green) and ADAMTS10 (red) in mouse dermal fibroblasts (MDFs), derived from wild-type mouse at postnatal day 3 (P3), yielded yellow signals in the merged images (suggesting co-localization) that were relatively rich in the peripheral and peri-nuclear regions (Fig. 2A, panel 4; Movie S1); this pattern is reminiscent of the RECK subcellular localization previously observed in NIH3T3 cells (Morioka et al., 2009). We also noted substantial heterogeneity in the levels of RECK expression among these MDFs (Fig. 2A, panel 2; note the green signals abundant in two of the several cells in this field). This approach, however, depends on the specificity of antibodies used (see Materials and Methods). We therefore took the second approach of co-expressing two proteins tagged with different fluorescent proteins, mRFP-RECK (red) and GFP-ADAMTS10 (green), in a human osteosarcoma cell line (MG-63) followed by confocal microscopy. This approach again yielded yellow signals rich in the peri-nuclear region (Fig. 2B; Movie S2).

We also performed *in situ* proximity ligation assays (PLA), which can detect close (<40 nm) co-localization between two molecules (Söderberg et al., 2006). PLA demonstrated close co-localization between endogenous RECK and ADAMTS10 in a mouse fibroblast-derived cell line, NIH3T3 (Fig. 2C,D). These results support the idea that RECK and ADAMTS10 may have chances to encounter and interact with each other under physiological conditions.

### Effects of RECK and ADAMTS10 on each other

**Fig. 3.**
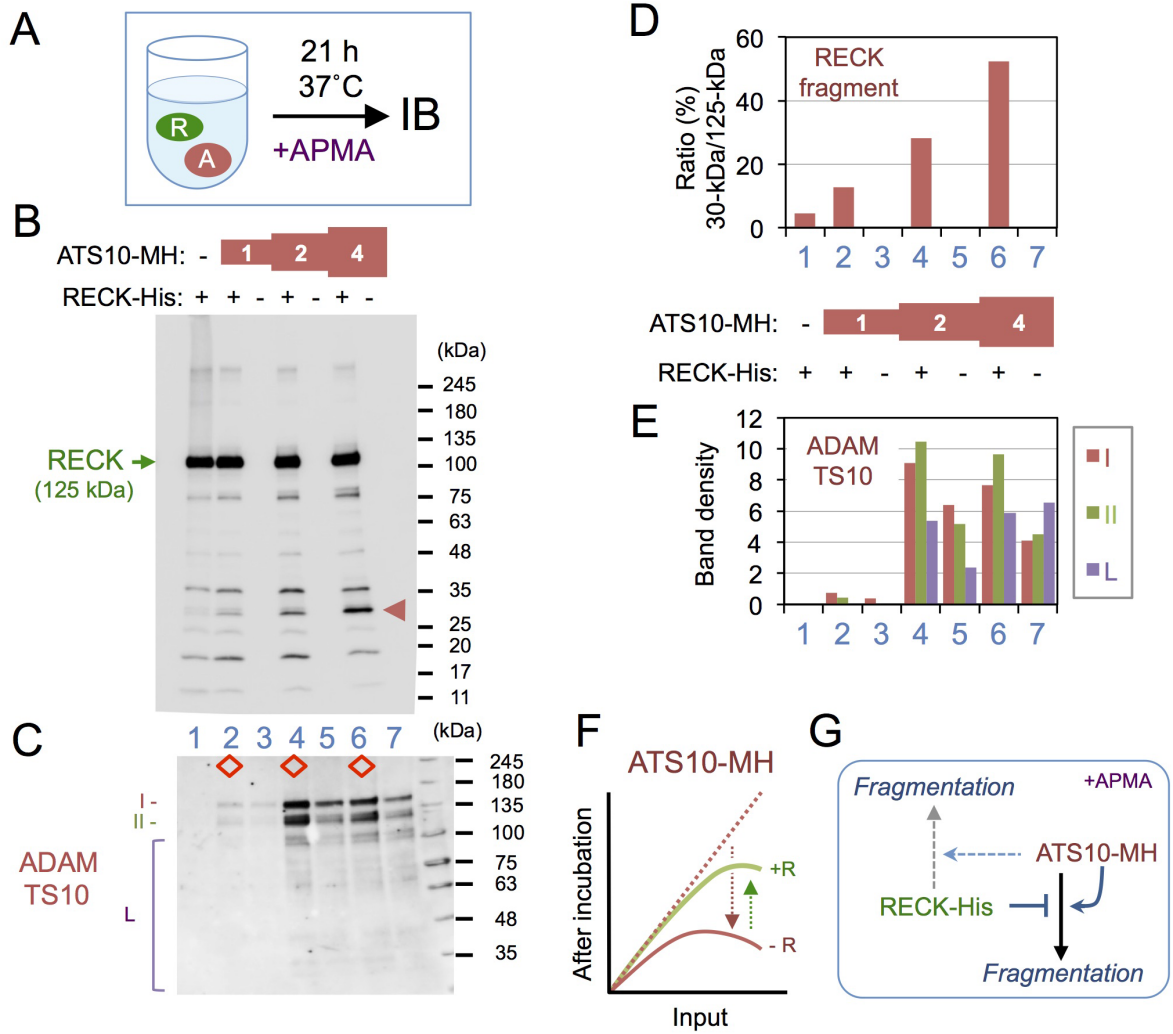
**Effects of RECK-His and ATS10-MH on each other.** (A) Experimental outline. ATS10-MH, at three different concentrations (relative concentration: 1, 2 and 4; see the section ‘Protein-mixing assay’ in Materials and Methods for detail), was incubated for 21 h without or with a constant amount of RECK-His in the presence of APMA, and the reaction mixtures were subjected to immunoblot assay (IB) using anti-RECK (B) or anti-ADAMTS10 (C). (D) Density of RECK fragment bands. Density of the full-length RECK-His (B, green arrow) and a fragment of ∼30 kDa (red arrowhead) in the blot shown in B were quantified using ImageJ, and their ratios are presented. (E) Density of ADAMTS10 bands. Relative intensities of a band or a group of bands in C, as indicated by the symbols (I,II and L), were quantified. The diamonds in C indicate samples containing RECK-His. See Fig. S3 for reproducibility and Fig. S4 for APMA- and time-dependence of the results in C. (F) Conceptual diagram to explain the results in panel C. X-axis: concentration of ATS10-MH before incubation. Y-axis: concentration of ATS10-MH after incubation. Red curve: ATS10-MH alone. Green curve: ATS10-MH with RECK-His. When incubated alone, the level of ATS10-MH declined at the higher concentration range (red arrow), suggesting dose-dependent autocatalytic fragmentation. In the presence of RECK-His, such decline was less prominent (green arrow), suggesting inhibition of ATS10 MH-fragmentation by RECK-His. (G) Summary of findings.

ADAMTS10 is a protease (Apte, 2009; Hubmacher and Apte, 2015); RECK has been studied as a protease inhibitor (Miki et al., 2007; Oh et al., 2001; Omura et al., 2009; Takahashi et al., 1998). We therefore performed simple mix-and-incubation experiments (Fig. 3A) to ask two obvious questions: (i) could ADAMTS10 cleave RECK? (ii) Could RECK inhibit ADAMTS10? In this experiment, we tried to activate pro-ADAMTS10 using p-aminophenyl mercuric acetate (APMA), a chemical known to activate several other members of the metazincin family.

When RECK-His (125 kDa) was incubated alone or in the presence of different amounts of ATS10-MH in buffer containing APMA for 21 h and then visualized by immunoblot assay, a dose-dependent increase in the density of ∼30 kDa fragment of RECK (Fig. 3B, arrowheads) was detected (Fig. 3D), although the decrease in full-length RECK band was not so prominent, suggesting that ADAMTS10 has a weak activity to cleave RECK.

A previous study by Kutz and colleagues (Kutz et al., 2011) also demonstrated that recombinant human ADAMTS10 gave rise to multiple bands in immunoblot assays and this phenomenon required its protease catalytic site (E393), suggesting that the phenomenon represented autocatalytic fragmentation. ATS10-MH (derived from mouse cDNA) shows two groups of prominent bands: one corresponding to its pro-form and the other close to the size of the mature form (I and II in Fig. 3C). When the amount of ATS10-MH was increased, the overall density of immunoblot bands increased up to a certain input protein concentration but did not increase at a higher concentration (Fig. 3C, compare lanes 3, 5 and 7; densitometric data in Fig. 3E, bars 3, 5 and 7). At the highest ATS10-MH concentration, the density of bands I and II decreased (Fig. 3E, bar 7 versus 5 in red and green) while the density of the smaller fragments increased (Fig. 3E, bars 7 versus 5 in purple), suggesting that ATS10-MH underwent dose-dependent autocatalytic fragmentation (illustrated by the downward arrow in Fig. 3F). In the presence of RECK-His, on the other hand, the density of ATS10-MH bands increased (Fig. 3C, compare lanes 2, 4 and 6 to lanes 3, 5 and 7; Fig. 3E, bars 2, 4 and 6 versus bars 3, 5 and 7), suggesting that RECK-His stabilizes ATS10-MH under these conditions (illustrated by the upward arrow in Fig. 3F). Similar results were obtained in another independent experiment (Fig. S3). The decrease in ATS10-MH band density at higher concentrations and the effect of RECK-His to yield higher ATS10-MH band density were clear only when APMA was added (Fig. S4B, lane 6 versus 3, lane 5 versus 6) and undetectable at an earlier time point (1.5 h; Fig. S4C, lane 5 versus 7, lanes 4 and 6 versus 5 and 7).

Taken together, these results indicate that ATS10-MH has a weak activity to cleave RECK-His and support the idea that RECK-His inhibits the autocatalytic fragmentation of ATS10-MH (illustrated in Fig. 3G).

### Effects of ATS10-MH on RECK function

**Fig. 4.**
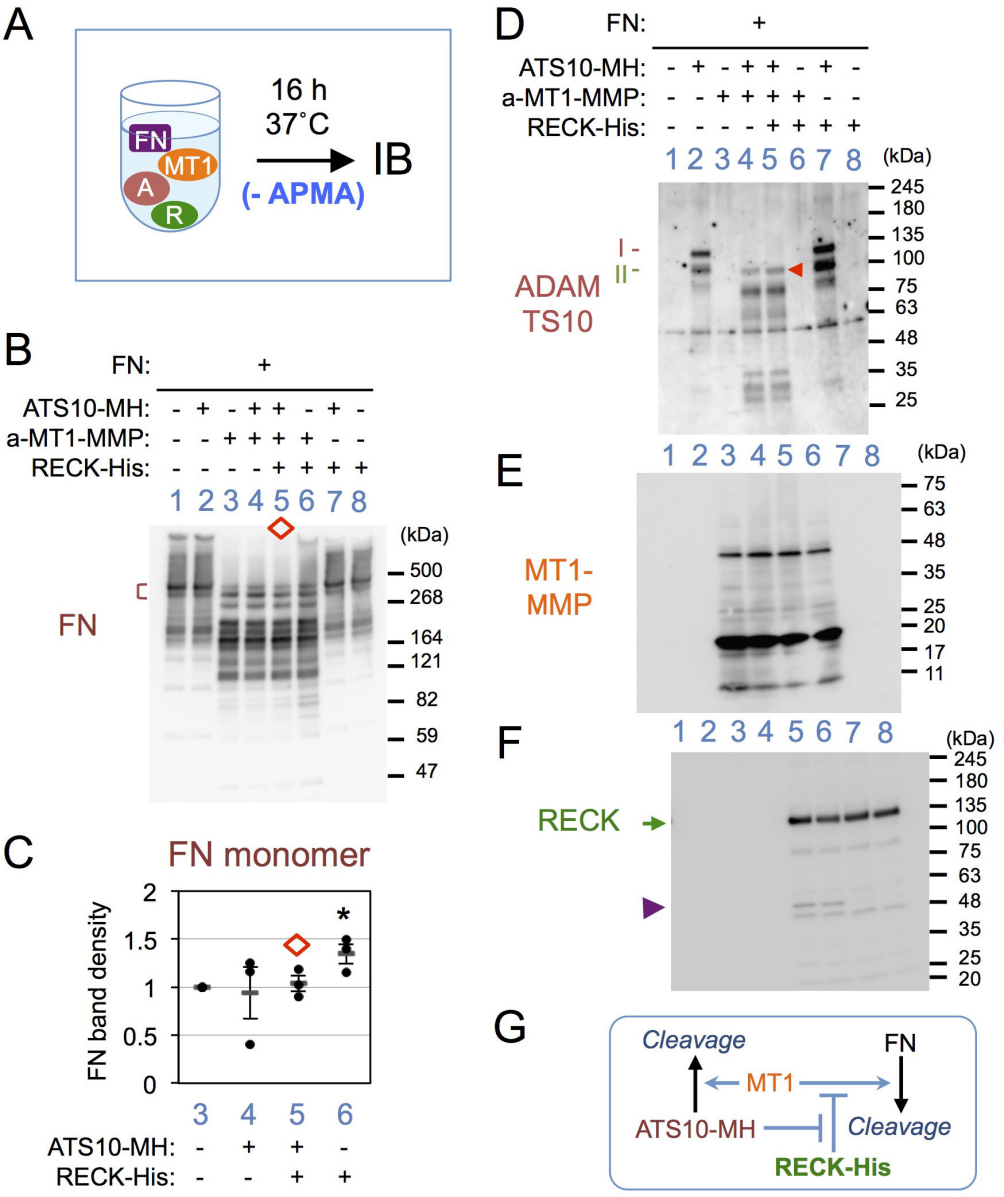
**Effects of RECK-His and ATS10-MH on FN-cleavage by MT1-MMP.** (A) Experimental outline. FN was mixed with ATS10-MH, MT1-MMP (furin-activated) and/or RECK-His in various combinations, incubated for 18 h, and the reaction mixture was subjected to immunoblot assay using anti-FN (B; non-reducing conditions), anti-ADAMTS10 (D; reducing conditions), anti-MT1-MMP (E; non-reducing conditions) or anti-RECK (F; reducing conditions). (C) Density of the monomer FN bands indicated by brown bracket in B. The data (mean±s.e.m.) from three independent experiments are shown. *P<0.05. Note that ATS10-MH enables MT1-MMP to cleave FN even in the presence of RECK-His (diamond in B and C), that an ATS10-MH band of ∼123 kDa remains after cleavage by MT1-MMP (arrowhead in D), that a RECK-His band of ∼50 kDa is produced by MT1-MMP (arrowhead in F), and that the intensity of the full-length RECK-His band is slightly higher when ATS10-MH is present (arrow in F). (F) Schematic summary of the results.

We next asked whether ATS10-MH affects the known function of RECK to inhibit MMPs by mix-and-incubation experiments with four purified factors (see Fig. 4A) in the absence of APMA. Furin-activated MT1-MMP cleaves fibronectin (FN) into smaller fragments as expected (Sternlicht and Werb, 2001) (Fig. 4B, lane 3 versus 1), and this reaction is inhibited by RECK-His (Fig. 4B, compare the bracketed region of lanes 6 and 3; Fig. 4C, 6 versus 3). In this system, ATS10-MH suppresses the effect of RECK-His (Fig. 4B, compare the bracketed region of lanes 5 and 6; Fig. 4C, lane 5 versus 6). One possibility is that ATS10-MH cleaves and reduces the level of intact RECK-His, allowing MT1-MMP to freely work again; however, RECK-His seems to be largely intact under these conditions (Fig. 4F, lane 5 versus 6). A more feasible model is that ATS10-MH binds RECK-His thereby interfering with the interaction between MT-MMP and RECK-His. This experiment also demonstrated the activity of MT1-MMP to efficiently cleave ATS10-MH (Fig. 4D, lane 4 versus 2). These findings are summarized in Fig. 4G.

### Effects of RECK on ADAMTS10 associated with the cells

**Fig. 5.**
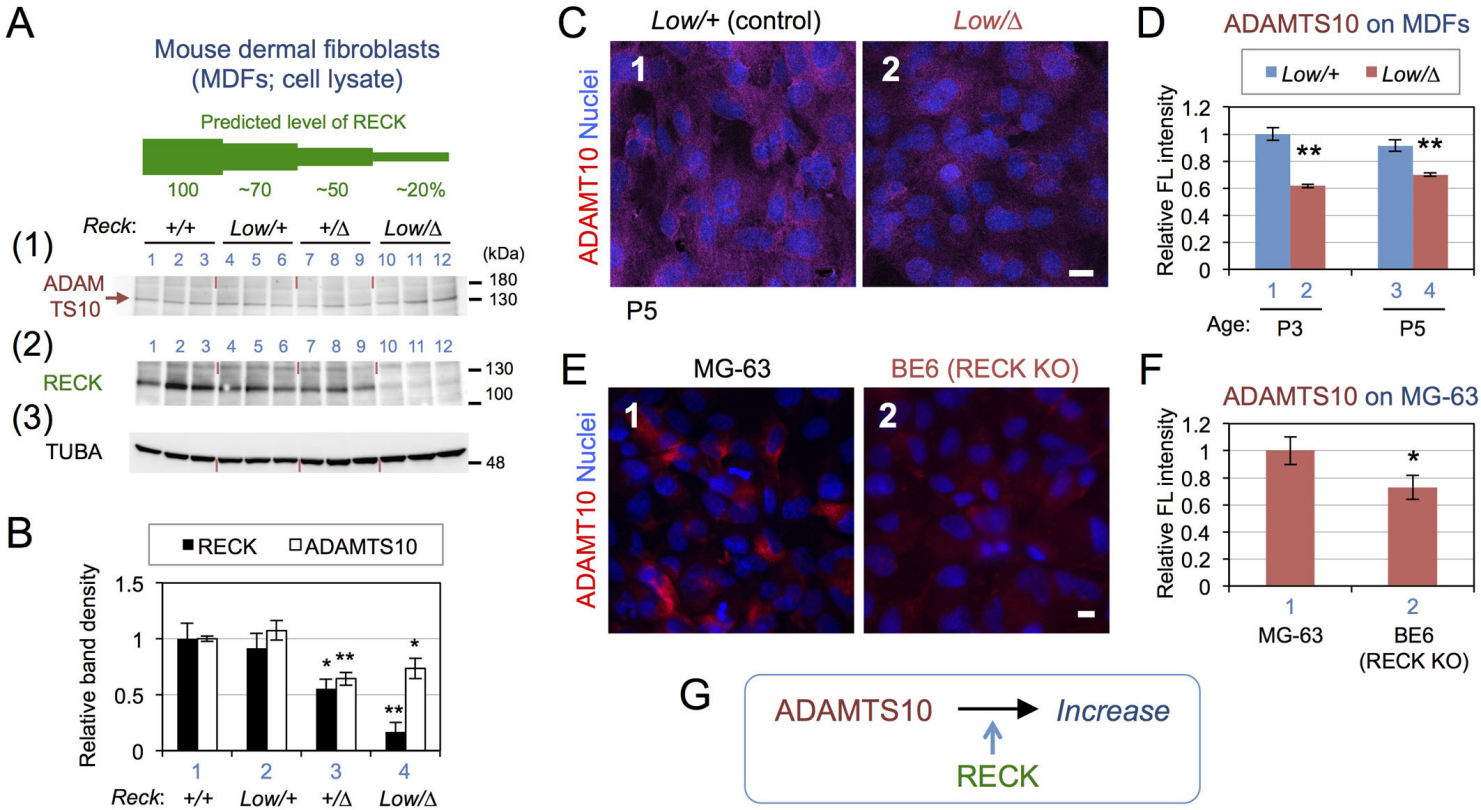
**Effects of reduced *Reck* expression on ADAMTS10 in cultured mesenchymal cells.** (A,B) Immunoblot assay with MDF cell lysates. Primary MDFs prepared from three animals of the indicated *Reck* genotypes were grown to confluence, and the cell lysates prepared from these cultures were subjected to immunoblot assay using anti-ADAMTS10 (A-1) and anti-RECK (A-2) antibodies, respectively; the latter blot was re-probed with anti-α-tubulin (TUBA) antibodies (A-3). The levels of RECK expression predicted from the genotype are schematically depicted in green above the blots. (B) Densitometry of the RECK band (black bar) shown in A-2 and the ADAMTS10 band (white bar) shown in A-1 (brown arrow). The value from each lane was normalized against that of TUBA and then divided by the mean value of +/+ samples. Bar represents mean±s.e.m. of three samples. *P<0.05, **P<0.01 (as compared to *+/+* MDFs). (C,D) Immunofluorescence staining of MDFs. MDFs prepared from *Low/*Δ and *Low/+* (control littermate) mice at P3 or P5 were stained with anti-ADAMTS10 antibodies (magenta) followed by nuclear counterstaining (blue) (C). Scale bar: 10 µm. Total fluorescence intensity of the images as shown in C (*n*=8) was determined, and the values (mean±s.e.m.) are presented as a bar graph in D. **P<0.01 (between the mutant and control littermates). (E,F) Immunofluorescence staining of MG-63 cells. MG-63 cells and its RECK-deficient derivative (BE6) were stained with anti-ADAMTS10 antibodies (magenta) followed by nuclear counterstaining (blue). Images were recorded using a fluorescence microscope (Zeiss Axioplan). Total fluorescence intensity of the images as shown in E (*n*=8) was determined using ImageJ and the values (mean±s.e.m.) are compared in F. *P<0.05. The levels of ADAMTS10 mRNA was not significantly different between these pairs of cells (data not shown). (G) Summary of findings.

In an attempt to confirm the biological relevance of the above findings using recombinant proteins, we prepared MDFs from mice with four different RECK expression levels: 100% (*+/+*), ∼70% (*Low/+*), ∼50% (*+/*Δ) and ∼20% (*Low/*Δ) (see Fig. S5A,B). Note that among these mice, conspicuous developmental abnormalities are found only in *Low/*Δ mice (Yamamoto et al., 2012) but more subtle phenotypes, especially after damage, have been found in *+/*Δ mice (Gutierrez et al., 2015; Wang et al., 2010). Confluent cultures of these cells (three animals per genotype) were lysed and subjected to immunoblot assay to detect RECK, ADAMTS10 and α-tubulin (Fig. 5A). The level of RECK in the cell lysates decreased in agreement with their genotypes (Fig. 5A, panel 2; densitometry data in Fig. 5B, black bars). When the level of cellular RECK protein was low (≤50%: *+/*Δ and *Low/*Δ), the level of ADAMTS10 associated with the cells was significantly reduced (Fig. 5B, white bars). Reduction of ADAMTS10 associated with MDFs derived from *Low/*Δ mice (P3 and P5) could also be detected by immunofluorescence staining (Fig. 5C,D). The level of ADAMTS10 associated with BE6, a RECK-deficient MG-63 clone, was significantly lower and diffuse compared to the parental line (Fig. 5E,F).

Taken together, our data indicate that RECK interacts with ADAMTS10 and increases the level of ADAMTS10 associated with the cells, possibly by protecting ADAMTS10 from degradation (Fig. 5G).

## DISCUSSION

Compelling evidence from experimental studies as well as analyses of clinical specimens implicate RECK in tumor suppression (Chung et al., 2012; Fakhry et al., 2016; Hill et al., 2011; Noda and Takahashi, 2007; Yoshida et al., 2012). Previous biochemical evidence led to the idea that competitive inhibition of MMPs may be the major molecular basis of RECK's biological functions. The affinity of RECK for MMPs (Ki∼10^−8^) (Miki et al., 2007; Oh et al., 2001; Omura et al., 2009; Takahashi et al., 1998), however, is lower than that of the prototypic endogenous MMP inhibitors, TIMPs (Ki: 10^−9^-10^−16^ M) (Bourboulia and Stetler-Stevenson, 2010; Butler et al., 1999; Murphy, 2011), raising the possibility that RECK may have additional molecular functions. Our attempts to find novel RECK-interactors using the unbiased approach of yeast two-hybrid screening led to the detection of another new target, ADAMTS10, a member of a metalloproteinase family distinct from MMPs.

Our experiments using recombinant proteins (i.e. ATS10-MH) demonstrated dose-dependent auto-degradation of ADAMTS10 (see Fig. 3). We speculate that the large variability in its RECK-binding activity observed at higher dosages in a solid-phase binding assay (e.g. Fig. 1E, 0.5 unit) might reflect such auto-degradation. In immunoblot assays, our preparations of ATS10-MH consistently yielded two sets of major bands of ∼138 kDa (band I) and ∼123 kDa (band II) (see Fig. 3C), and band II is likely to contain activated ADAMTS10 (Somerville et al., 2004). Of note, RECK-His not only increases the intensity of ATS10-MH but also increases the relative intensity of band II to band I (see Fig. 3C; e.g. compare lane 6 with lane 7), raising the possibility that RECK may help generate and selectively stabilize the active form of ADAMTS10. Such cooperative interaction is in sharp contrast to the effect of RECK on MMPs (i.e. negative regulation) and is consistent with our observation that RECK and ADAMTS10 promote fibrillin microfibril formation in a cooperative manner in cultured cells (T. Matsuzaki et al., unpublished).

Yeast two-hybrid assays indicated that RECK has the potential to bind to a C-terminal portion, rather than the catalytic domain, of ADAMTS10 (Fig. 1B–D). Ihara and Nishiwaki reported that a nematode ADAMTS protein, MIG-17, undergoes autocatalytic activation which requires its C-terminal PLAC domain and suggested that this domain might interact with the metalloproteinase domain to promote autocatalytic activation (Ihara and Nishiwaki, 2007). Of note, the PLAC domain of MIG-17 shows the highest similarity to that of mammalian ADAMTS10 among all family members (Ihara and Nishiwaki, 2007). We therefore speculate that RECK may facilitate this interaction while inhibiting the auto-degradation of once activated ADAMTS10. The reason why this interaction requires Zn^2+^ is presently unclear. Metalloproteinases are known to coordinate Zn^2+^ in their catalytic domain, and therefore Zn^2+^ may well have allosteric effects on other domains. Alternatively, Zn^2+^ may be required for this particular protein-protein interaction more directly.

Some of our data demonstrated a weak, APMA-dependent activity of ADAMTS10 to cleave RECK in a dose-dependent manner (Fig. 3B). An activity of ADAMTS10 to suppress the MMP-inhibiting activity of RECK was also found (Fig. 4B,C). In these respects, ADAMTS10 may be considered as a regulator of RECK's functions.

Previous studies by Apte and colleagues (Kutz et al., 2008; Somerville et al., 2004) raised several important questions about the molecular nature of ADAMTS10, which include (1) why ADAMTS10 undergoes extensive fragmentation, (2) why a large fraction of ADAMTS10 forms aggregates in the culture supernatant, and (3) why its pro-domain cannot be efficiently cleaved off by furin. These questions are based mainly on the observations made using the epithelial cell line HEK293F which expresses a minimal level of RECK protein. Our findings that MT1-MMP efficiently cleaves pro-ADAMTS10 yielding a fragment of about the size of its active form (see Fig. 4D) and that RECK influences the fragmentation of activated ADAMTS10 (see Fig. 3C,E–G) shed some new light on these questions. In mouse embryos, RECK expression is relatively high in mesenchymal cells in which ADAMTS10 is also expressed abundantly (Somerville et al., 2004). The fate of ADAMTS10 co-produced with RECK by mesenchymal cells is likely to differ from that of ADAMTS10 produced by HEK293F cells. Whether such difference has to do with the reported differences in the modes of microfibril formation between mesenchymal and epithelial cells (Baldwin et al., 2014; Cain et al., 2016) is an interesting issue to be addressed in future studies.

In summary, this study has uncovered the novel ability of RECK to interact with ADAMTS10, an enzyme known to be critical for connective tissue development, and to increase the amount of ADAMTS10 associated with the cell. This interaction may also affect the function of RECK itself, a tumor suppressor downregulated in a wide variety of cancers.

## MATERIALS AND METHODS

### Antibodies

Rabbit polyclonal anti-ADAMTS10 antibodies (pAb-867) were raised against a synthetic oligopeptide (SGHSKLPKRQRAC; in the second TSR domain). Other primary antibodies: mouse monoclonal antibodies against RECK (5B11D12) (Takahashi et al., 1998), FN (610078, BD), MT1-MMP (ab51074; Abcam) and α-tubulin (DM1A; Calbiochem/Merck Millipore, Burlington, USA). Secondary antibodies: anti-rabbit IgG-HRP (ab6721; Abcam), anti-mouse IgG-HRP (A4416; Sigma-Aldrich), anti-mouse IgG-CF488 (20018; Biotium, Fremont, USA), anti-mouse IgG-CF405M (20182, Biotium) and anti-rabbit IgG-CF647 (20282: Biotium). Antibody dilution: primary 1:1000 and secondary 1:20000 for immunoblot assays, primary 1:300 and secondary 1:1000 for immunofluorescence staining. Specificity of pAb-867 and 5B11D12 in immunofluorescence staining was assessed in MG-63 cells genetically manipulated to suppress (ADAMTS10) or inactivate (RECK) the expression of endogenous target genes (see Fig. S2).

### Reck mutant mice

The *Reck* mutant allele, Δ (also known as *Reck^tm2.2Noda^*) and *Low* (also known as *Reck^tm1.2Noda^*), have been described (de Almeida et al., 2015; Chandana et al., 2010; Yamamoto et al., 2012) and were on the *Mus musculus* C57BL/6 background. The animal experiments were approved by the Animal Experimentation Committee, Kyoto University and conducted in accordance with its regulations.

### DNA construction

A mouse embryo (E12, C57BL/6) cDNA library (2×10^6^ clones) was generated using the pGADT7 vector. Lambda Zap II-based cDNA libraries were also generated using the same cDNA pool and adult mouse brain cDNAs. A fragment of mouse *Reck* cDNA (codons 27-942; termed RF) was amplified by PCR (Table S2A), digested with EcoRI and SalI and inserted into pGBKT7 to obtain pGBKT7-mRECK. A full-length *Adamts10* cDNA was re-screened from a Lambda Zap II-based library; its upstream sequence (gcccctATGgct) was modified to make it better fit with the Kozak sequence (gaattcgccATGgct), and it was inserted between the EcoRI and XbaI sites of the pEF6/*Myc*-His A vector to obtain pEF6A-mAdamts10. To generate the vector expressing ATS10-MH, pEF6A-mAdamts10 was modified so that the termination codon of *Adamts10* was replaced in frame with the sequence encoding the MYC-His_6_ epitope tag present in the pEF6/*Myc*-His A vector. For protein production, the same coding sequence was inserted into pTet-splice (Tet-off) to obtain pTet-mAdamts10MycHis. For the yeast two-hybrid assay, an *Adamts10* cDNA (codons 25-1104; termed AF) was amplified by PCR (Table S2A), digested with EcoR1 and Sal I and inserted into pGBKT7 to obtain pGBKT7-mAdamts10. To generate cDNAs encoding deletion mutants of RECK or ADAMTS10, the target sequences were amplified by PCR and inserted into pGADT7 (Rd1-9 and Ad1-6). Sources of vectors and hosts: pGBKT7 and pGADT7 (Clontech, Palo Alto, USA), pEF6/*Myc*-His A (Invitrogen), lambda Zap II (Stratagene, La Jolla, USA), pTet-splice (GIBCO) and the yeast strain AH109 (Clontech). pLXSB-hRECK has been described (Morioka et al., 2009; Sasahara et al., 1999). To express EGFP-tagged ADAMTS10 protein in mammalian cells, the Ad1 cDNA was inserted into pEGFP-C1 (Clontech) containing the RECK signal peptide sequence (Morioka et al., 2009) to obtain p-ss-EGFP-Adamts10. pmRFP-RECK was also described previously (Morioka et al., 2009).

### Yeast two-hybrid assay

AH109 cells were transformed with pGBKT7-mRECK and selected on SD plates without tryptophan to obtain the indicator strain AH109-mRECK. To screen RECK-interactors (Table S1), AH109-mRECK cells were transformed with the pGADT7-based cDNA library and selected on SD plates without leucine, tryptophan, adenine, or histidine (-LTAH) and with 10 mM 3-amino-1,2,4-triazole (3AT; an inhibitor of histidine biosynthesis which increases the stringency of selection in a dose-dependent manner) for 11 days. For binding assays, the AH109 cells harboring pGBKT7-mAdamts10 or pGBKT7-Ad4 were transformed with pGADT7-RECK (RF) or its deletion mutants (Rd1-9), and their growth on SD (-LTAH) plates with 3AT at different concentrations (0, 2, 5, 10) was examined after incubation for 4 days (Fig. 1A,C). Similarly, AH109-mRECK cells were transformed with pGADT7-mAdamts10 (AF) or its deletion mutants (Ad1-6), and their growth on SD (-LTAH) plates with or without 3AT was examined.

### Cell culture and gene transfer

HEK293, MG-63 (ATCC) and NIH3T3 cells (a gift from Dr Robert H. Bassin) were maintained in growth medium (GM) consisting of DMEM supplemented with 10% fetal bovine serum (FBS), 100 µg/ml streptomycin sulfate and 100 U/ml penicillin G. MDFs were prepared from mouse-back skin (P3-P5) using the cold trypsin method (Freshney, 1987). Transfection was performed using Lipofectamine 2000 (Invitrogen), FuGENE 6 (Roche) or CalPhos Mammalian Transfection Kit (Clontech).

### Recombinant ATS10-MH protein

HEK293 Tet-off Advanced (Clontech) was stably co-transfected with pTet-mAdamts10MycHis and pEF6/*Myc*-His A, selected with 8 µg/ml blasticidin in GM (selection medium), and a clone abundantly producing ATS10-MH was maintained in selection medium containing 2 μg/ml doxycycline. Subconfluent cultures of these cells were washed with PBS and incubated in CD293 medium for 5 days. Culture supernatant was centrifuged to remove cellular debris, dialyzed against PBS, adjusted to 0.5 M NaCl and applied to a His-Trap HP column (GE) that was equilibrated with 20 mM imidazol. After washing with 40 mM imidazole, proteins were eluted using a step-gradient of imidazole. The 60 mM imidazol fraction rich in ATS10-MH was used in the solid-phase binding assay after replacing its solvent with 20 mM Hepes (pH7.2), 150 mM NaCl by ultrafiltration (AmiconUltra 50K; Millipore). The 250 mM imidazole fraction was dialyzed against 0.15 M NaCl/20 mM KPO_4_ (pH 8.0) and applied to a cation-exchange column (RESOURCE S, GE). The flow-through fraction was applied to an anion-exchange column (RESOURCE Q, GE); the column was washed with 20 mM KPO_4_ (pH 8.0) containing 310 mM NaCl, and the bound proteins were eluted sequentially using buffer containing 400 mM NaCl and 1 M NaCl, respectively. The 400 mM NaCl fraction was used for the protein-mixing assays shown in Fig. 4 and Fig. S4B. This fraction was further applied to a gel filtration column (Hiload 26/60) to eliminate small molecules and used for surface plasmon resonance assay. The 1 M NaCl fraction was dialyzed against 20 mM Hepes (pH 7.0)/180 mM NaCl and applied to a heparin HP column (GE), and the flow-through fraction was used for the protein-mixing assays shown in Fig. 3 and Fig. S4D.

### Solid-phase binding assay

Purified RECK-His protein (Omura et al., 2009) (100 ng/170 µl/well) was immobilized onto 96-well Maxisorp Plates (Nunc/Thermo Fisher Scientific, Waltham, USA) with 15 mM Na_2_CO_3_, 35 mM NaHCO_3_, 6 mM azide. The wells were blocked with 1% bovine serum albumin (200 µl), washed with PBS twice, and then with 20 mM Hepes pH7.4/150 mM NaCl (HBS). ATS10-MH (after His-Trap purification from 400 ml culture supernatant which yielded 3.2 ml buffer-exchanged sample – see the section ‘Recombinant ATS10-MH protein’ for details – 1 unit in Fig. 1E correspond to undiluted sample) serially diluted in HBS containing 2 mM CaCl_2_, 2 mM MgCl_2_, 2 nM ZnCl_2_ was added to the wells (170 µl/well) and incubated overnight at 4°C. After extensive washing with PBS containing 0.0005% Tween 20 (WB), ATS10-MH bound to the well was labeled with anti-ADAMTS10 and then with HRP-conjugated anti-rabbit IgG (Cell Signaling Technology), and detected using chemiluminescent HRP substrate (Immobilon Substrate, WBKLS0500, Millipore) followed by luminometry (MicroLumatPlus, PerkinElmer, Waltham, USA).

### Localization of RECK and ADAMTS10 in cultured cells

For immunofluorescence staining, wild-type MDFs were fixed in acetone at −80°C and stained with anti-ADAMTS10 (pAb-867; CF647) (Fig. 5C,D) or double-stained with anti-ADAMTS10 (pAb-867; CF647) and anti-RECK (5B; CF488) (Fig. 2A). For co-expression of fluorescent-protein-tagged proteins (Fig. 2B), MG-63 cells on 2-well chamber slides (SCS-002, Matsunami, Osaka, Japan) were co-transfected with p-ss-EGFP-Adamts10 (1 µg/well) and pmRFP-RECK (32) (1 µg/well) using Lipofectamine2000 and incubated for 2 days. Fluorescence signals were recorded using a confocal microscope (Leica sp8).

### *In situ* proximity ligation assay

Confluent NIH3T3 cells in 8-well chamber slides (8CS; SCS-008, Matsunami) were fixed with IHC Zinc Fixative (BD Biosciences) for 4.5 h and subjected to PLA using Duolink Green (Sigma-Aldrich) with anti-RECK (5B) and/or anti-ADAMTS10 (pAb-867) as primary antibodies and anti-Mouse PLUS and anti-Rabbit MINUS as secondary antibodies. PLA signals (green) and the number of nuclei (DAPI-stained) were quantified using ImageJ (NIH).

### Protein detection

Immunoblot assay was performed as described (Oh et al., 2001) using α-tubulin as a control. Cell lysate was prepared using lysis buffer containing 50 mM Tris pH7.5, 0.5 M NaCl, 1% NP40, 0.1% SDS, 0.5% deoxycholate, 5 mM N-ethylmaleimide and 20×protease inhibitor cocktail (03969, Nakarai, Kyoto, Japan). Samples were frozen at −80°C and just before SDS-PAGE, boiled for 10 min in Laemmli sample buffer (1×) containing 83 mM DTT. Images were captured using LAS3000 (Fujifilm, Tokyo, Japan). Densitometry was carried out using MultiGauge v3.2 (Fujifilm) or ImageJ (NIH).

### Protein-mixing assay

To test the effects of RECK and ADAMTS10 on each other (Fig. 3, Figs S3 and S4), purified RECK-His (0.36 pmol), ATS10-MH (multiple concentrations; ‘1’=0.72 pmol), or both in 20 µl Buffer E [150 mM NaCl, 5 mM CaCl_2_, 2.5 mM MgCl_2_, 0.5 mM ZnCl_2_, 0.05% Brij-35, 20 mM Tris.HCl (pH 7.0), with or without 1 mM APMA] were incubated at 37°C for the indicated period of time. To test the effects on FN (Fig. 4; Fig. S4), mature MT1-MMP (0.31 pmol), RECK-His (0.48 pmol) and/or ATS10-MH (0.43 pmol) were mixed in various combinations with FN (0.87 pmol) and incubated at 37°C for the indicated period of time in 40 µl Buffer E. The reaction mixture was separated by SDS-PAGE under non-reducing or reducing conditions. To prepare mature MT1-MMP, recombinant MT1-MMP (Millipore; CC1043, 230 ng) was pre-treated for 1 h with 20 ng furin in 62 µl Buffer E.

### Statistics

Significance of difference between two groups of data was evaluated using Student's *t*-test.

## Supplementary Material

Supplementary information

First Person interview

## References

[BIO033985C1] Apte S. S. (2009). A disintegrin-like and metalloprotease (reprolysin-type) with thrombospondin type 1 motif (ADAMTS) superfamily: functions and mechanisms. *J. Biol. Chem.* 284, 31493-31497. 10.1074/jbc.R109.052340 19734141 PMC2797218

[BIO033985C2] Baldwin A. K., Cain S. A., Lennon R., Godwin A., Merry C. L. and Kielty C. M. (2014). Epithelial-mesenchymal status influences how cells deposit fibrillin microfibrils. *J. Cell Sci.* 127, 158-171. 10.1242/jcs.134270 24190885 PMC3874785

[BIO033985C3] Bourboulia D. and Stetler-Stevenson W. G. (2010). Matrix metalloproteinases (MMPs) and tissue inhibitors of metalloproteinases (TIMPs): positive and negative regulators in tumor cell adhesion. *Semin. Cancer Biol.* 20, 161-168. 10.1016/j.semcancer.2010.05.002 20470890 PMC2941566

[BIO033985C4] Butler G. S., Hutton M., Wattam B. A., Williamson R. A., Knauper V., Willenbrock F. and Murphy G. (1999). The specificity of TIMP-2 for matrix metalloproteinases can be modified by single amino acid mutations. *J. Biol. Chem.* 274, 20391-20396. 10.1074/jbc.274.29.20391 10400663

[BIO033985C5] Cain S. A., Mularczyk E. J., Singh M., Massam-Wu T. and Kielty C. M. (2016). ADAMTS-10 and -6 differentially regulate cell-cell junctions and focal adhesions. *Sci. Rep.* 6, 35956 10.1038/srep35956 27779234 PMC5078793

[BIO033985C6] Chandana E. P., Maeda Y., Ueda A., Kiyonari H., Oshima N., Yamamoto M., Kondo S., Oh J., Takahashi R., Yoshida Y. et al. (2010). Involvement of the Reck tumor suppressor protein in maternal and embryonic vascular remodeling in mice. *BMC Dev. Biol.* 10, 84 10.1186/1471-213X-10-84 20691046 PMC2923112

[BIO033985C7] Chung T. T., Yeh C. B., Li Y. C., Su S. C., Chien M. H., Yang S. F. and Hsieh Y. H. (2012). Effect of RECK gene polymorphisms on hepatocellular carcinoma susceptibility and clinicopathologic features. *PLoS ONE* 7, e33517 10.1371/journal.pone.0033517 22428065 PMC3299798

[BIO033985C8] Dagoneau N., Benoist-Lasselin C., Huber C., Faivre L., Megarbane A., Alswaid A., Dollfus H., Alembik Y., Munnich A., Legeai-Mallet L. et al. (2004). ADAMTS10 mutations in autosomal recessive Weill-Marchesani syndrome. *Am. J. Hum. Genet.* 75, 801-806. 10.1086/425231 15368195 PMC1182109

[BIO033985C9] de Almeida G. M., Yamamoto M., Morioka Y., Ogawa S., Matsuzaki T. and Noda M. (2015). Critical roles for murine Reck in the regulation of vascular patterning and stabilization. *Sci. Rep.* 5, 17860 10.1038/srep17860 26658478 PMC4675993

[BIO033985C10] Duckert P., Brunak S. and Blom N. (2004). Prediction of proprotein convertase cleavage sites. *Protein Eng. Des. Sel.* 17, 107-112. 10.1093/protein/gzh013 14985543

[BIO033985C11] Faivre L., Gorlin R. J., Wirtz M. K., Godfrey M., Dagoneau N., Samples J. R., Le Merrer M., Collod-Beroud G., Boileau C., Munnich A. et al. (2003). In frame fibrillin-1 gene deletion in autosomal dominant Weill-Marchesani syndrome. *J. Med. Genet.* 40, 34-36. 10.1136/jmg.40.1.34 12525539 PMC1735272

[BIO033985C12] Fakhry A. B., Ahmed A. I., AbdelAlim M. A. and Ramadan D. I. (2016). RECK gene promoter rs10814325 polymorphism in egyptian patients with hepatocellular carcinoma on top of chronic hepatitis C viral infection. *Asian Pac. J. Cancer Prev.* 17, 2383-2388. 27268601

[BIO033985C13] Freshney R. I. (1987). Disaggregation of the tissue and primary culture. In *Culture of Animal Cells: a Manual of Basic Technique*, pp. 107-126. New York: Wiley-Liss.

[BIO033985C14] Gutierrez J., Droppelmann C. A., Contreras O., Takahashi C. and Brandan E. (2015). RECK-mediated beta1-integrin regulation by tgf-beta1 is critical for wound contraction in mice. *PLoS ONE* 10, e0135005 10.1371/journal.pone.0135005 26247610 PMC4527692

[BIO033985C15] Hill V. K., Ricketts C., Bieche I., Vacher S., Gentle D., Lewis C., Maher E. R. and Latif F. (2011). Genome-wide DNA methylation profiling of CpG islands in breast cancer identifies novel genes associated with tumorigenicity. *Cancer Res.* 71, 2988-2999. 10.1158/0008-5472.CAN-10-4026 21363912

[BIO033985C16] Hubmacher D. and Apte S. S. (2015). ADAMTS proteins as modulators of microfibril formation and function. *Matrix Biol.* 47, 34-43. 10.1016/j.matbio.2015.05.004 25957949 PMC4731137

[BIO033985C17] Ihara S. and Nishiwaki K. (2007). Prodomain-dependent tissue targeting of an ADAMTS protease controls cell migration in Caenorhabditis elegans. *EMBO J.* 26, 2607-2620. 10.1038/sj.emboj.7601718 17491590 PMC1888677

[BIO033985C18] Kutz W. E., Wang L. W., Dagoneau N., Odrcic K. J., Cormier-Daire V., Traboulsi E. I. and Apte S. S. (2008). Functional analysis of an ADAMTS10 signal peptide mutation in Weill-Marchesani syndrome demonstrates a long-range effect on secretion of the full-length enzyme. *Hum. Mutat.* 29, 1425-1434. 10.1002/humu.20797 18567016

[BIO033985C19] Kutz W. E., Wang L. W., Bader H. L., Majors A. K., Iwata K., Traboulsi E. I., Sakai L. Y., Keene D. R. and Apte S. S. (2011). ADAMTS10 protein interacts with fibrillin-1 and promotes its deposition in extracellular matrix of cultured fibroblasts. *J. Biol. Chem.* 286, 17156-17167. 10.1074/jbc.M111.231571 21402694 PMC3089559

[BIO033985C20] Miki T., Takegami Y., Okawa K., Muraguchi T., Noda M. and Takahashi C. (2007). The reversion-inducing cysteine-rich protein with Kazal motifs (RECK) interacts with membrane type 1 matrix metalloproteinase and CD13/aminopeptidase N and modulates their endocytic pathways. *J. Biol. Chem.* 282, 12341-12352. 10.1074/jbc.M610948200 17329256

[BIO033985C21] Morioka Y., Monypenny J., Matsuzaki T., Shi S., Alexander D. B., Kitayama H. and Noda M. (2009). The membrane-anchored metalloproteinase regulator RECK stabilizes focal adhesions and anterior-posterior polarity in fibroblasts. *Oncogene* 28, 1454-1464. 10.1038/onc.2008.486 19169281

[BIO033985C22] Muraguchi T., Takegami Y., Ohtsuka T., Kitajima S., Chandana E. P. S., Omura A., Miki T., Takahashi R., Matsumoto N., Ludwig A. et al. (2007). RECK modulates Notch signaling during cortical neurogenesis by regulating ADAM10 activity. *Nat. Neurosci.* 10, 838-845. 10.1038/nn1922 17558399

[BIO033985C23] Murphy G. (2011). Tissue inhibitors of metalloproteinases. *Genome Biol.* 12, 233 10.1186/gb-2011-12-11-233 22078297 PMC3334591

[BIO033985C24] Noda M. and Takahashi C. (2007). Recklessness as a hallmark of aggressive cancer. *Cancer Sci.* 98, 1659-1665. 10.1111/j.1349-7006.2007.00588.x 17725805 PMC11158385

[BIO033985C25] Noda M., Vallon M. and Kuo C. J. (2016). The Wnt7's Tale: a story of an orphan who finds her tie to a famous family. *Cancer Sci.* 107, 12924 10.1111/cas.12924 PMC4970824 26934061

[BIO033985C26] Oh J., Takahashi R., Kondo S., Mizoguchi A., Adachi E., Sasahara R. M., Nishimura S., Imamura Y., Kitayama H., Alexander D. B. et al. (2001). The membrane-anchored MMP inhibitor RECK is a key regulator of extracellular matrix integrity and angiogenesis. *Cell* 107, 789-800. 10.1016/S0092-8674(01)00597-9 11747814

[BIO033985C27] Omura A., Matsuzaki T., Mio K., Ogura T., Yamamoto M., Fujita A., Okawa K., Kitayama H., Takahashi C., Sato C. et al. (2009). RECK forms cowbell-shaped dimers and inhibits matrix metalloproteinase-catalyzed cleavage of fibronectin. *J. Biol. Chem.* 284, 3461-3469. 10.1074/jbc.M806212200 19022775

[BIO033985C28] Sasahara R. M., Takahashi C. and Noda M. (1999). Involvement of the Sp1 site in ras-mediated downregulation of the RECK metastasis suppressor gene. *Biochem. Biophys. Res. Commun.* 264, 668-675. 10.1006/bbrc.1999.1552 10543990

[BIO033985C29] Shimoda M., Principe S., Jackson H. W., Luga V., Fang H., Molyneux S. D., Shao Y. W., Aiken A., Waterhouse P. D., Karamboulas C. et al. (2014). Loss of the Timp gene family is sufficient for the acquisition of the CAF-like cell state. *Nat. Cell Biol.* 16, 889-901. 10.1038/ncb3021 25150980

[BIO033985C30] Söderberg O., Gullberg M., Jarvius M., Ridderstråle K., Leuchowius K.-J., Jarvius J., Wester K., Hydbring P., Bahram F., Larsson L.-G. et al. (2006). Direct observation of individual endogenous protein complexes in situ by proximity ligation. *Nat. Methods* 3, 995-1000. 10.1038/nmeth947 17072308

[BIO033985C31] Somerville R. P. T., Jungers K. A. and Apte S. S. (2004). Discovery and characterization of a novel, widely expressed metalloprotease, ADAMTS10, and its proteolytic activation. *J. Biol. Chem.* 279, 51208-51217. 10.1074/jbc.M409036200 15355968

[BIO033985C32] Sternlicht M. D. and Werb Z. (2001). How matrix metalloproteinases regulate cell behavior. *Annu. Rev. Cell Dev. Biol.* 17, 463-516. 10.1146/annurev.cellbio.17.1.463 11687497 PMC2792593

[BIO033985C33] Takahashi C., Sheng Z., Horan T. P., Kitayama H., Maki M., Hitomi K., Kitaura Y., Takai S., Sasahara R. M., Horimoto A. et al. (1998). Regulation of matrix metalloproteinase-9 and inhibition of tumor invasion by the membrane-anchored glycoprotein RECK. *Proc. Natl. Acad. Sci. USA* 95, 13221-13226. 10.1073/pnas.95.22.13221 9789069 PMC23764

[BIO033985C34] Vanhollebeke B., Stone O. A., Bostaille N., Cho C., Zhou Y., Maquet E., Gauquier A., Cabochette P., Fukuhara S., Mochizuki N. et al. (2015). Tip cell-specific requirement for an atypical Gpr124- and Reck-dependent Wnt/beta-catenin pathway during brain angiogenesis. *Elife* 4, e06489 10.7554/eLife.06489 PMC4456509 26051822

[BIO033985C35] Wang H., Imamura Y., Ishibashi R., Chandana E. P. S., Yamamoto M. and Noda M. (2010). The Reck tumor suppressor protein alleviates tissue damage and promotes functional recovery after transient cerebral ischemia in mice. *J. Neurochem.* 115, 385-398. 10.1111/j.1471-4159.2010.06933.x 20796170

[BIO033985C36] Yamamoto M., Matsuzaki T., Takahashi R., Adachi E., Maeda Y., Yamaguchi S., Kitayama H., Echizenya M., Morioka Y., Alexander D. B. et al. (2012). The transformation suppressor gene Reck is required for postaxial patterning in mouse forelimbs. *Biol. Open* 1, 458-466. 10.1242/bio.2012638 23213437 PMC3507216

[BIO033985C37] Yoshida Y., Ninomiya K., Hamada H. and Noda M. (2012). Involvement of the SKP2-p27(KIP1) pathway in suppression of cancer cell proliferation by RECK. *Oncogene* 31, 4128-4138. 10.1038/onc.2011.570 22158033

