# Supplementary material for: The RECK tumor-suppressor protein binds and stabilizes ADAMTS10
Source: Biol Open. 2018 Oct 15;7(10):bio033985. doi: 10.1242/bio.033985 (PMC6215420; doi:10.1242/bio.033985)
Supplement: Supplementary information [file biolopen-7-033985-s1.pdf]

© 2018. Published by The Company of Biologists Ltd | Biology Open (2018): doi:10.1242/bio.033985: Supplementary information

FIRST PERSON

First person – Tomoko Matsuzaki

First Person is a series of interviews with the first authors of a selection of papers published in Biology Open, helping early-career researchers promote themselves alongside their papers. Tomoko Matsuzaki is first author on 'The RECK tumor-suppressor protein binds and stabilizes ADAMTS10', published in BiO. Tomoko is an assistant professor in the lab of Makoto Noda at Kyoto University, Kyoto, Japan, investigating the interaction between the microenvironment and cancer cells.

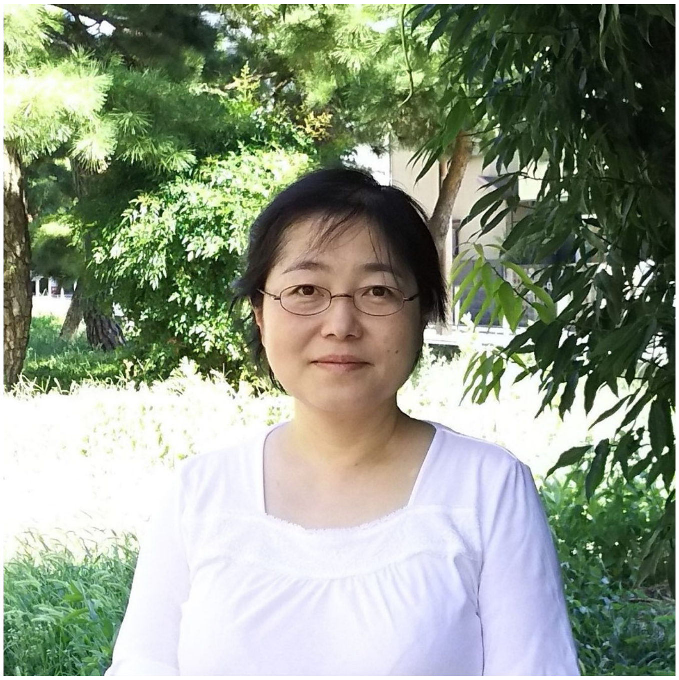
Tomoko Matsuzaki

What is your scientific background and the general focus of your lab?

After graduation from college, I worked as a lab technician for several years at RIKEN and the Cancer Research Foundation, and then got my PhD degree in medical science in Dr Yoji Ikawa's laboratory (Department of Retroviral Regulation) in Tokyo Medical and Dental University. I also worked with Dr Motoya Katsuki in a venture company, working to make knockdown mice using shRNA. Subsequently, I worked with Dr Yoshio Miki in a genome project and then with Dr Tetsuo Noda, trying to generate several mutant mice. Since moving to Kyoto, I have been working on a very interesting but obscure protein named RECK. Our group focuses on elucidating the functions of RECK, hoping to utilize the knowledge and materials in future diagnoses and therapies.

How would you explain the main findings of your paper to non-scientific family and friends?

In many common cancers, such as those in the lung, colon, pancreas and prostate, the amount of RECK is reduced as compared to healthy cells. When we artificially express RECK in cancer cells, their malignant behaviors, such as invasion and metastasis, are suppressed in animal models. We found that another protein, ADAMTS10, is a binding partner of RECK. ADAMTS10 has been linked to a totally different type of disease called Weill–Marchesani syndrome (WMS). WMS is a rare genetic disorder of connective tissue, characterized by short stature, eye abnormalities, short fingers and joint stiffness. Many of these symptoms are opposite to those of Marfan syndrome (MS), another more common connective tissue disorder. ADAMTS10 is inactivated in WMS. We found in this study that binding with RECK stabilizes ADAMTS10. Our findings suggest that cancer and WMS might involve some common molecular machinery.

What are the potential implications of these results for your field of research?

The only known functions of RECK were to inhibit several matrix metalloproteinases and to stimulate WNT signaling, but we felt that these could not explain all the phenotypes of Reck-deficient mice, which show mid-gestation lethality with reduced tissue integrity and defects in vascular and neural development. The discovery of a new binding partner, ADAMTS10, has opened up a new avenue toward better understanding how RECK works and how ADAMTS10 is regulated. ADAMTS10 has been implicated in the formation of an elastic fiber made of a large molecule called fibrillin-1 (FBN1). Mutations in FBN1 have been linked to both WMS and MS. Thus, it is likely that RECK has some functional connection to FBN1 and may also be implicated in MS. It would be a challenge and pleasure for us to solve the secrets of the mysterious relationship between these connective tissue disorders and cancer.

What has surprised you the most while conducting your research?

In this study, we purified recombinant ADAMTS10 and investigated it using basic protein chemistry techniques. The biggest surprise was that when I incubated ADAMTS10 in a series of different concentrations and detected it by immunoblot assay, the band intensity did not reflect the amount of input protein. In the first run, I suspected that I had made a mistake. However, the results were reproducible in my subsequent, careful experiments, and we came to the conclusions that ADAMTS10 digests itself at higher concentrations and that RECK slows this self-digestion. Another surprise was my initial failure in the SPR assay using these proteins. After a struggle, I found a specific requirement of Zn^{2+} for the RECK–ADAMTS10 interaction. It could be that Zn^{2+} is required for one or both of these proteins to take the conformation required for the interaction. In nature, a drop in extracellular Zn^{2+} concentration may free these proteins and alter their influence on the ECM.

What, in your opinion, are some of the greatest achievements in your field and how has this influenced your research?

In the field of cancer research, the success of immune checkpoint inhibitors in therapy is a major breakthrough. The use of exosomes

Tomoko Matsuzaki's contact details: Department of Molecular Oncology, Kyoto University Graduate School of Medicine, Yoshida-Konoe-cho, Sakyo-ku, Kyoto 606-8501, Japan.
E-mail: matsuzaki.tomoko.7w@kyoto-u.ac.jp

Biology Open (2018): doi:10.1242/bio.033985: Supplementary information

in cancer diagnosis and therapy may also be an interesting topic. In the general bioscience field, CRISPR/Cas9 is a great gene-editing technology that was used to knock out RECK in a human cell line in this study. We utilized another great achievement of our time, the use of green fluorescent protein (GFP) for molecular imaging, to show co-localization of RECK and ADAMTS10 around the cells.

"Some new findings of little immediate utility today may provide valuable seeds for future scientific development."

What changes do you think could improve the professional lives of early-career scientists?

Some new findings of little immediate utility today may provide valuable seeds for future scientific development. I hope that policy makers realize this fact and decide to allocate more funding for basic science and to take a bottom-up approach. I also feel that reporting such naïve but original discoveries of potential importance in scientific journals is getting harder. This may reflect the increase in the number of researchers or specialized fields, but I think another important factor is the attitude (and philosophy) of peer reviewers and editors toward science. Competition can often be a driving force in scientific research. Our goal, however, is not to win the competition or to show off the abundance of personal knowledge but to advance, synthesize and enrich the knowledge of humankind. I think early-career scientists should be trained to become excellent, constructive reviewers.

What's next for you?

I hope to better understand the relationship between the ECM and cancer. It would be nice if we could take advantage of our experience with RECK and find something useful in cancer medicine.

Reference

Matsuzaki, T., Kitayama H., Omura, A., Nishimoto, E., Alexander, D. B. and Noda, M. (2018). The RECK tumor-suppressor protein binds and stabilizes ADAMTS10. *Biol. Open* **7**: bio033985.
